# Involuntary Career Changes: A Lonesome Social Experience

**DOI:** 10.3389/fpsyg.2022.899051

**Published:** 2022-06-02

**Authors:** Jonas Masdonati, Caroline É. Frésard, Michaël Parmentier

**Affiliations:** ^1^Research Center in Vocational Psychology and Career Counseling, Institute of Psychology, University of Lausanne, Lausanne, Switzerland; ^2^Psychological Sciences Research Institute, Catholic University of Louvain, Louvain-la-Neuve, Belgium

**Keywords:** career transition, career change, thematic analysis, relational influences, social barriers, social resources, loneliness

## Abstract

Like any other career process, career changes are influenced by relationships. Moreover, involuntary career changes are a challenging, yet understudied, career transition. Based on a relational perspective of work and careers, we investigated the way people’s social environment affects the process and experience of involuntary career changes. Specifically, we aimed to identify the sources of relational influences and to understand how these influences affect career changes. Semi-structured interviews were carried out with 14 adults who were forced to change career because of unemployment or health issues. Through thematic analysis, we identified three sources of relational influences (personal, work, and institutional environment) and three forms of influence that others had on career changes (positive, negative, and ambivalent). These influences manifested at four distinct moments of the process: When participants were leaving their former job, when they were shifting between their former occupation and a new livelihood, when they were exploring new career options, or when they were trying to implement their new career plan. Overall, results suggest that involuntary career changes are deeply shaped by heterogeneous and differentiated relational influences. The effect of the personal environment varied depending on the moment of the career change process. In particular, family and friends tended to be perceived as barriers when it came to shifting from the old to a new occupation and implementing a new career plan. The work environment mostly had a negative effect on the career change experience, suggesting the labor market might be somewhat refractory toward adult career changers. Institutions played a critical role throughout the change process, with support structures often being perceived as inappropriate, but with guidance professionals generally recognizing participants’ difficulties. Moreover, diverse forms of ambivalence characterized the identified relational influences, which were sometimes both appreciated and avoided or had ambiguous and fluctuating effects. Finally, although being a fundamentally social experience, involuntary career changes were also characterized by moments of loneliness that reflected the inadequacy of available support and a sense of shame associated with the status of career changer. Study limitations, research perspectives, and practical implications at the labor market, institutional, and individual levels are addressed.

## Introduction

### Involuntary Career Changes

During their working lives, people tend to go through several career transitions, which scholars have argued are on the rise in recent years (Fouad and Bynner, 2008; Rudisill et al., 2010; Urbanaviciute et al., 2019). Career transitions are defined as “moves across different types of boundaries” that may represent “both minor discontinuities and major interruptions in an individual career” (Chudzikowski, 2012, p. 298). Some transitions are highly normative and expected (e.g., the transition to retirement), whereas others are rather non-normative and unanticipated (e.g., demotions) and can involve higher discontinuities. In addition, career transitions can be of several types (Heppner and Scott, 2006): entry or reentry transitions (e.g., the passage from school to work or labor market reintegration after a break), maintenance transitions (e.g., a role change within the same company or occupation), advancement transitions (e.g., a promotion toward a better position), and leave-or-seek transitions (e.g., a change of occupational sector). The latter are also called career or occupational changes and they imply a shift to a new occupation that is not in line with the previous occupation (Ibarra, 2006; Carless and Arnup, 2011; Peake and McDowall, 2012). While research on normative transitions and upward mobility is abundant, career changes have been less studied (Sullivan and Al Ariss, 2021).

Depending on the reasons and outcomes of this process, career changes can represent both opportunities to grow and perturbing periods in workers’ careers (Carless and Arnup, 2011; Chudzikowski, 2012; Masdonati et al., 2017). Intentionality or willingness is a key dimension that tends to differentiate the experience of career changes (Heppner and Scott, 2006; Fouad and Bynner, 2008; Stuart et al., 2009; Zacher, 2017). In general, workers who voluntarily decide to change their career seek an improvement in their working (e.g., job satisfaction) or personal life (e.g., work-life balance). In contrast, other workers change job or occupation involuntarily, which might indicate a loss of control over their careers and lead to situations of uncertainty and decreased work and life satisfaction (Bernhard-Oettel and Näswall, 2015). Surprisingly, although voluntary career changes have been extensively studied (e.g., Négroni, 2007; Barclay et al., 2011; Howes and Goodman-Delahunty, 2014), little is known about the challenges of involuntary career changes (Peake and McDowall, 2012). Moreover, the rare studies on this topic focus on specific populations, such as veterans (e.g., Haynie and Shepherd, 2011; Kulkarni, 2020) and athletes (e.g., Arvinen-Barrow et al., 2018). The experiences of people who were forced to change career, such as workers with health issues (e.g., Baldridge and Kulkarni, 2017) or those who change occupational sector because of lack of job opportunities in their initial occupation (e.g., Bernhard-Oettel and Näswall, 2015), are then relatively undocumented.

### Career Changes as Relational Experiences

Career changes have been addressed through various theoretical lenses (Baruch and Sullivan, 2022), such as the chaos theory of careers (Peake and McDowall, 2012), the life-span, life-space approach (Barclay et al., 2011), the life-course perspective (Howes and Goodman-Delahunty, 2014), a psychosocial transitional perspective (Masdonati et al., 2017), the stress-coping approach (Rudisill et al., 2010), and the boundaryless career perspective (Chudzikowski, 2012). Most of these theoretical frameworks refer to a social dimension that might affect career development processes. Nevertheless, the social dimension is often reduced to a background factor. Research employing these theoretical lenses mostly investigated the ways people’s social environment contributed to the decision to change career. For example, Howes and Goodman-Delahunty (2014) showed that police officers and teachers initiated a career change because they felt unvalued and lacked social recognition. Professional opportunities stemming from social connections prompted occupational changes for mid-career individuals in Peake and McDowall (2012). More in line with the present article, Motulsky (2010) appears to be one of the few studies that have thoroughly examined how an individual’s social environment intervenes in the transition process beyond the decision to change career. It showed that women’s voluntary midlife career changes were shaped by an articulation of social connections and disconnections from parents, the partner, friends, colleagues, and supervisors. However, none of these studies examined how social influences affect involuntary career change processes.

In parallel, broader theoretical approaches to career development (e.g., Brown and Lent, 2017; McMahon and Patton, 2019) increasingly emphasize the impact of contextual and social environmental factors on career paths, though in polarized and rather static ways (i.e., in terms of proximal and distal supports and barriers, see Sheu and Bordon, 2017). Kenny et al. (2018) proposed the relational perspective on work and careers to address the influences that different types of relationships can have on careers and to articulate the work and non-work challenges of people who struggle in their working lives. Rooted, among other things, in the earlier writings of Blustein (2011) and Flum (2015), this perspective is structured around four tenets. First, “Work is a vehicle for human connection” (Kenny et al., 2018, p. 138), meaning that working is a fundamentally relational experience and that relationships at work can provide social connection and meaning in life, but can also be detrimental for individuals’ well-being. Second, “Family and other close relationships are vital domains of life experience that interact with work in reciprocal and complex ways” (p. 139). This tenet highlights that events at work affect life outside work (particularly one’s family), and vice versa, and that this reciprocal influence can be either positive or negative. Third, “Relationships, both current and those internalized from past experience, affect the career development process and trajectory” (p. 139). In that sense, relationships can be both limiting (i.e., consist of barriers to one’s career development) or positive (i.e., function as emotional or instrumental support). This tenet also suggests that a temporal dimension is to be considered when studying relational influences on careers: Past relational experiences affect the way people cope with current challenges at work and develop throughout their careers. Fourth, “Culture, social marginalization, and economic status exert critical roles in shaping work and relational experiences; their meaning; and the dynamics between work, family, and community life and opportunities and outcomes” (p. 139). This last tenet advocates a broad conceptualization of relationships, which are not limited to direct interactions with the proximal social environment, but also include cultural and societal processes. Thus, people also relate with the society they live in, including institutions, support structures, and the labor market.

When applied more specifically to involuntary career changes, the relational perspective on work and careers (Kenny et al., 2018) implies considering the extent to which relationships at work play a role in the career change processes and outcomes (Tenet 1). It also suggests that relationships outside the work sphere possibly affect—and are affected by—career changes (Tenet 2). Moreover, the relational perspective suggests considering social supports and barriers from a temporal viewpoint, and particularly the influence of internalized past experiences on current career changes (Tenet 3). Finally, a broad conceptualization of relationships implies understanding the ways both proximal (e.g., co-workers and family) and distal factors (e.g., institutions and labor market) affect career changes (Tenet 4).

### The Current Study

In sum, while voluntary work transitions are well documented, research is needed to understand the process and experiences of involuntary career changes. This is especially crucial because involuntary transitions are more likely to represent disruptive events for individuals as they are generally less anticipated, thus leaving less time and space for individuals to develop their resources and prepare for the transition compared to voluntary transitions (Fouad and Bynner, 2008; Blustein et al., 2013; Sullivan and Al Ariss, 2021). In particular, given the relational nature of careers (Blustein, 2011; Flum, 2015), it is of pivotal importance to study the influence of others on people’s experiences of a career change. Research on relational processes during voluntary career change has suggested that this influence needs to be considered in a dynamic and nuanced way (Motulsky, 2010). Accordingly, the general aim of the present study was *to understand how others influence the process and experience of involuntary career changes*. Based on the relational perspective on work and careers (Kenny et al., 2018), this general aim was divided into two specific research goals. The first specific goal was *to identify the sources of relational influences on involuntary career change processes and experiences*. These sources can be deployed both within and outside the working sphere as well as at both proximal and distal levels. Our second specific goal was *to understand how relational influences affect the process of involuntary career changes*. The role of others is to be considered through a temporal perspective, which means studying the way both anticipated future relationships and internalized past relationships influence current experiences of career change.

The current study was carried out in the French-speaking part of Switzerland, representing 23% of 8.5 million Swiss habitants. Similar to several other countries, workers in Switzerland show increasing occupational mobility intentions and behaviors (Office Fédéral de la Statistique [OFS], 2020). For example, between 2018 and 2019, one out of five workers quit their job. In the same period, 4.6% apparently left their jobs involuntarily, either because of health issues, firings, or expiring contracts. However, the proportion of this occupational mobility that involved a career change is not clear. From a statistical viewpoint, involuntary career changes seem an overlooked phenomenon or an issue that is difficult to quantify and grasp. Based on these statistics, the present study focused on the two populations who seem at greater risk of experiencing involuntary career changes, namely workers changing jobs because of health issues and people who are looking for a job in a new occupational sector because of a lack of opportunities in their initial occupational field. These two populations can benefit from state support, either through the unemployment or disability public scheme. The eligibility for state support for implementing a career change and the extent and type of support depend on the assessment of each beneficiary’s situation, provided by career professionals such as job coaches and career counselors.

## Materials and Methods

### Paradigmatic Position

To address our study aims, we implemented an inductive qualitative research design based on thematic analyses of semi-structured interviews with involuntary career changers. Inductive qualitative research is indeed considered an appropriate method to study recent changes in the world of work (Pratt and Bonaccio, 2016). According to the classification of qualitative research paradigms suggested by Ponterotto (2005), the present study can be qualified as postpositivist-constructivist. Indeed, our research objectives, data collection, and analysis strategies suggest that while recognizing the uniqueness of each participant’s story, we assume the existence of a shared reality that is common to people living the same situation. Specifically, on the one hand, the research can in part be described as postpositivist, since it adopted semi-structured interviews with key questions asked to all participants, involved reaching consensus among coders, and considered category frequencies when presenting and discussing findings. On the other hand, our study is also constructivist in that interviews were partly adjusted to each participant’s unique experiences and the interviewer-interviewee interaction, researchers shared reflections on their feelings and experiences after each interview, and participants’ narratives and voices prevailed over frequencies when reporting and discussing results.

### Participants and Procedure

Fourteen participants, seven men and seven women, aged 29–58 (*M* = 40.36, *SD* = 8.84), who were engaged in an involuntary career change because of unemployment (*N* = 6) or health issues (*N* = 8), took part in the study. As indicated in Table 1, the occupations in which they had previously worked were mainly in the service sector (except for one carpenter) and covered a variety of occupational fields (e.g., security, health, transportation, and sales) and educational requirements, ranging from vocational training (e.g., hairdresser) to university (e.g., surgeon). Eight participants were of Swiss origin, two were binational, and four came from other European countries. Inclusion criteria required having begun a career change recently and involuntarily because of either physical or mental health problems or lack of employment opportunities in their occupational sector.

**TABLE 1:** Demographics.

Name	Age	Gender	Origin	Reason for career change	Previous occupation	Career plan
William	41	M	Swiss/United Kingdom	Unemployment	Flight coordinator	Unknown
Nancy	41	F	Swiss/United Kingdom	Unemployment	Librarian	Educational project manager
Marie	42	F	Swiss	Unemployment	Bookseller	Librarian
Sarah	45	F	Swiss	Unemployment	Executive assistant	HR manager
Louis	46	M	Belgian	Unemployment	Airline CEO	Business executive
Henry	58	M	Swiss	Unemployment	Humanitarian coordinator	Unknown
Beatriz	29	F	Swiss	Health	Saleswoman	Secretary
Kevin	29	M	French	Health	Hairdresser	HR assistant
Frédéric	29	M	Swiss	Health	Carpenter	Architectural draftsman
Jean	31	M	Swiss	Health	Money transporter	Security manager
Gabriel	34	M	French	Health	Carpenter	Office technician
Veronique	44	F	Swiss	Health	Hairdresser	Administrative employee
Giuliana	47	F	European*^a^*	Health	Surgeon	Judge
Anna	49	F	Swiss	Health	Nurse	Care coordinator

*Pseudonyms used.*

*^a^Participant has requested not to disclose her country of origin.*

The recruitment procedure began with presenting the project to public and parapublic institutions in the cantons of Vaud, Genève, and Neuchâtel (French-speaking regions of Switzerland) whose mission is to coach adults forced to change career and reintegrate them into the labor market. We then asked the collaborators of the 13 institutions that had accepted participation in the study (mainly job coaches and career counselors) to invite users who matched the inclusion criteria to participate in a research interview. The contact details of the interested users were referred to the researchers, who contacted them by e-mail to set up an interview. All interviews were conducted remotely using Zoom software, which is considered appropriate for this type of data collection (Archibald et al., 2019). Interviews lasted between 34 and 146 mins (*M* = 92), they were entirely recorded and transcribed, and they were carried out by three researchers, a professor, a Ph.D. student, and a final-year master’s student in vocational psychology and career counseling. Since the interviews could sometimes generate strong emotions, at the end of each interview the interviewer took a moment to acknowledge these emotions and restore, if necessary, the initial emotional state. This was facilitated by the fact that all the interviewers are trained in counseling psychology. Participants who needed additional support were referred to appropriate services. Upon completion of each interview, the interviewer wrote a note summarizing the interviewee’s situation and their main challenges and proposed a self-analysis.

Participation in the study was voluntary and was not rewarded. The interview itself was considered an incentive to participate in the research as it could provide participants with a valuable moment to reflect on their life and career paths and plans. The research was carried out in line with the American Psychological Association’s ethical standards and the study procedure was approved by the ethics committee of the Faculty of Social and Political Sciences of the University of Lausanne (Project Number C_SSP_052021_00003).

### Interview Guideline

The interview guideline was divided into seven sections. The first section gathered participants’ sociodemographic information. In the second section, interviewees were asked to describe their career paths (e.g., “Can you tell me what jobs you have held throughout your career, and how long they lasted?”). The third section focused on the process and experience of career change (e.g., “Can you tell me what brought you to this point?”, “What are the reasons for your career change?”, “How do you feel about changing professions/leaving your profession/entering a new profession?”). The fourth interview section investigated personal, social, and vocational identity processes (e.g., “How does the career change also change how you see yourself as a person?”, “How do you talk about this situation to people around you?”, “Try to project yourself into the distant future, for example, 10 years from now. Who do you think you will be at that moment professionally?”). Personal and social resources and barriers were explored in the fifth section (e.g., “What resources facilitate your coping in facing the challenges of a career change?” and “What is standing in your way?”). Finally, Sections 6 and 7 covered participants’ relationship to work and, when pertinent, to training (e.g., “How has your career change affected the importance you attach to work in your life and the meanings you attribute to it, if at all?”).

The complete guideline is provided in the Appendix. Interview questions were formulated *ad hoc* for a broader research project that aims to understand what characterizes involuntary career change processes through the concepts of career shock (Akkermans et al., 2018), identity work (Kulkarni, 2020), and relationship to work (Fournier et al., 2020) and training (Kaddouri, 2011). For the present study, we essentially focused on participants’ discourse related to the third, fourth, and fifth sections.

### Analyses

Data analysis was carried out by two researchers, a professor and a junior researcher in vocational psychology and career counseling. According to Morrow (2005), the researchers’ “horizons of understanding” (p. 250) influence reflexive processes during the analysis stage and have to be made explicit through a researcher-as-instrument statement. The professor has experienced several career and geographical transitions but has never gone through an authentic career change. The junior researcher has experienced a voluntary career change from teacher to career counselor. Having both been trained as career counselors, the two researchers are familiar with the realities of people who face a career change. A third researcher, a senior researcher in the same field, was external auditor. His role was to provide a critical perspective on the analyses and the study as a whole, to help find consensus when needed, and to prevent possible power issues. Analyses were performed with the software MAXQDA, and the six-step procedure for reflexive thematic analysis suggested by Braun and Clarke (2006) was followed.

#### Familiarizing With the Data

One researcher went through the transcriptions and identified all the interview passages where participants referred to relational aspects of their career change experience or indicated the presence of others in the career change process. The selected passages were divided into two parts and each researcher carefully read one of the two parts. They then met to generate initial ideas about interesting aspects to be further analyzed. The researchers reached a consensus and organized the data analysis around three *structuring themes*: the *type* of influence others have on the career change process and experience (in line with the second specific research question); the *sources* of relational influences (in line with the first specific research question); and the *moment* when the relational influences took place (in line with the temporal dimension addressed in the theoretical framework, see Kenny et al., 2018).

#### Generating Initial Codes

The two researchers interchanged their parts and each researcher coded half of the retained transcriptions. The codes were data-driven, and each code was classified according to the three structuring themes. The two researchers then met a second time to share and compare their respective initial codes.

#### Searching for Themes

During the same meeting, the researchers sorted their codes and collated them into overarching themes. Concretely, they gathered codes referring to the same structuring theme and then combined and grouped them into *themes*. The following themes were identified: Positive, negative, and ambivalent influences were the themes within the structuring theme “type of influence”; personal environment, work environment, institutional influences, and societal influences were the themes within the structuring theme “source of influence;” and past, present, and anticipated influences were the themes within the structuring theme “moment of influence.”

#### Reviewing Themes

During a third meeting, the two researchers reviewed the themes to ensure they accurately reflected the data set and satisfactorily covered the study objectives. Two modifications were implemented at this stage. First, within the source of influence structuring theme, we merged the themes of institutional and societal influences because coding these two sources separately was sometimes impossible. Second, we divided the moment of influence structuring theme into four temporal themes, namely “leaving” (the former occupation), “shifting” (the change process), “exploring” (planning a new career), and “implementing” (the new career plan). This separation seemed to us to be better aligned with the actual stages of career change reported by our participants. A fourth meeting was then organized to identify subthemes consensually. To provide a holistic and temporal perspective on our results, we crossed all the themes as well as compared and collated the codes within each of these intersections. For example, subthemes were created to describe more precisely positive influences coming from the personal environment and occurring when participants were leaving the former occupation, and so on. At this stage, some codes were removed because they did not refer to the study objectives or were exclusive to a single participant, and maps of the structuring themes, themes, and subthemes were designed and discussed.

#### Defining and Naming Themes

**FIGURE 1:**
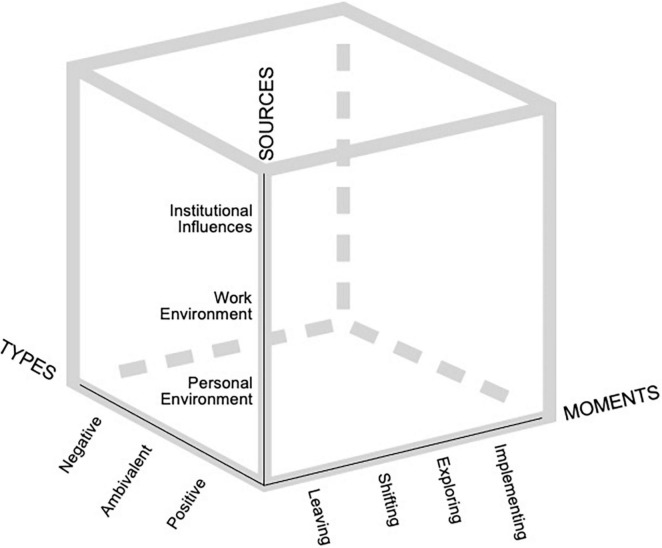
Visual representation of the structuring themes (all caps) and themes.

One researcher defined and described all the structuring themes, themes, and subthemes and returned to the data to identify examples of quotes that illustrate each subtheme. These definitions, descriptions, and quotes were revised and validated by the second researcher with the goal of identifying compelling labels and relevant, informative excerpts. Final minor adjustments were implemented on the subthemes. The structuring themes and themes are illustrated in Figure 1, whereas the “Results” section addresses each subtheme.

#### Producing the Report

This final step consisted of transforming the findings from a multidimensional configuration to a linear narrative. We hierarchized the structuring themes and organized the “Results” section according to our research goals, prioritizing our understanding of how others influence the process and experience involuntary career changes, and depending on whether they facilitate, hinder, or bring ambivalence to the career change. Thus, findings were organized first around the type of influence structuring theme, then around the source of influence structuring theme, and finally around the moment structuring theme.

### Trustworthiness

In line with Morrow’s (2005) suggestion concerning trustworthiness criteria for postpositivist-constructivist qualitative research, we ensured our study credibility, transferability, dependability, and confirmability. Reflexive notetaking right after each interview and the use of the shared perspectives of two researchers enhanced the credibility of the analyses. The explanation of the context and the limits of the study (proposed in the “Introduction” section and at the end of the discussion, respectively) enable us to estimate the transferability of our results. Finally, the detailed description of the analysis procedure, the clarification of the two researchers’ horizons of understanding through a researcher-as-instrument statement, and the contribution of an external auditor should ensure the dependability and confirmability of the study.

## Results

We identified three structuring themes in the initial analysis steps (Figure 1). The first structuring theme was the type of influence that others had on participants, which we divided into three themes: positive influences, referring to others perceived as resources and facilitators of the career change process; negative influences, indicating others perceived as obstacles or constraints; and ambivalent influences, others perceived either as both a resource and an obstacle, or having an unclear, ambiguous effect on the career change experience. The second structuring theme was the source of influence, which we divided into three themes: the personal environment, covering influences from family members and friends; the work environment, referring to influences from the professional context, such as colleagues and other members of participants’ companies; and institutional influences, indicating influences from the institutions with which participants were in contact. This category also included people from the society in which they evolved more broadly. The third structuring theme was the moment when the influence manifested or the phase within the career change process. We identified four key moments, yielding four additional themes: leaving—the period when participants learned that they needed to change careers while still in their first occupation; shifting, referring to the in-between phase in which they dealt with the challenges of transitioning “from a before to an after;” exploring, when participants focused on and were concerned with their future career plans; and implementing, which covers the concrete preparation and implementation of their career changes to new occupations.

In the following sections, we present the subthemes that emerged within these themes. We first divided the subthemes according to the type of influence. Within each type of influence, we then organized them according to the source of influence. Finally, for each source of influence, we present subthemes chronologically (i.e., according to the moment when they occur).

### Positive Relational Influences

As indicated in Table 2, 10 subthemes allowed us to qualify positive relational influences on participants’ career change experiences.

**TABLE 2:** Subthemes within positive relational influences.

	Leaving	Shifting	Exploring	Implementing
Personal environment	Socioemotional support (9) Instrumental support (5)		Socioemotional support and role models (5)	
Work environment	Discovery of a new career option (3)			
Institutions	Acknowledgment of the issue (4)	Instrumental support (money and time) (4) Professionals who make the person feel unique (4) Psychological support (3)	Help with career decision strategies (6)	Support for professional integration (6)

*Numbers refer to frequencies of participants.*

#### Positive Influences From the Personal Environment

Three subthemes covered positive influences coming from the personal environment. Participants stressed the importance of both instrumental and socioemotional support during the period when they prepared to leave their former occupations. For example, William, 41, a former flight coordinator who lost his job, mentioned socioemotional support:

There are also moments that are lower, more difficult. And then, as we said, you have to share with your friends—you have to talk about it, you have to discuss it; it’s very important. […] It starts again and then the positive comes back, there is the motivation to move forward.

Support from friends and family was also highlighted during the exploring period, again taking the form of socioemotional help (e.g., encouragement) but also role models. Frédéric, 29, a former carpenter suffering from disabling back pain, talked about a friend who had gone through a career change and inspired him:

Most of my friends, they all studied, except for one who also did a career change. He used to be an optician, then he said that it wasn’t really his values and he decided to change, and now he’s in his last year of ergotherapy. […] I’ve always admired him for his career change, his desires, his values. And it’s true that I decided—given that this year wasn’t great and all, and that I had the opportunity to be able to do it—I said to myself, “Well, this year I’m the one who decides to change job and change professions, make this career change.”

#### Positive Influences From the Work Environment

Participants reported one positive influence on their career changes coming from the company they were about to leave. They were indeed able to identify new career options (e.g., an appealing new occupation or an integration opportunity) while still in the former job. Stating that allergies prevented him from continuing working as a hairdresser, Kevin, 29, identified the human resource sector as a promising career alternative. Before leaving his job, he had already decided to enroll in a training program in this field:

I said to myself: “OK, what could suit me?” And I remembered that […] my boss was really, let’s say artistic director, but on the other hand the management of the hairdressing salon and the employees were a bit handed to me. So, if we had to hire an apprentice, I was the one who had to do the necessary steps for the recruitment, for his training, to manage the holidays of my employees. […] I thought it could be nice to have a bit of a human resources side. So, I did a little research and […] I decided to get a certificate in personnel management on the side of my job as a hairdresser.

#### Positive Influences From Institutions

Institutional influences operated as resources throughout the four career change moments and manifested in six subthemes. Some participants appreciated that their health issues were already acknowledged during their former work experiences, which facilitated the process of leaving. Frédéric was able to access public disability insurance before leaving his job, which helped him look forward to the future:

Now, I’m still employed at my old company, so I’m on sick leave. The disability insurance accepted that I do an early career change. Because all my medical files noted that I could do it, and now I’m looking for a new apprenticeship, and as soon as I start, the disability insurance will support me financially.

Three additional forms of institutional supports were mentioned during the shifting phase. First, some participants stressed the importance of the instrumental support they accessed from state disability or unemployment insurance, both in terms of financial resources and time to reflect on and cope with the career change. Second, interviewees mentioned that the professionals they interacted with had been able to make them feel unique and step away from the profiteering image that is often ascribed to beneficiaries of public disability or unemployment schemes. Third, other participants benefited from psychological help. Beatriz, 29, a former saleswoman, clearly valued the fact that her counselor made her feel special in terms of motivation:

My counselor is awesome. I mean, he’s seen me as a different person. Because people who go to the disability insurance, in general they don’t give themselves as much means, they don’t do as much research to be able to get out of it. Whereas he saw in me a really optimistic side, saying to myself, “today I can’t, but tomorrow I can.” And for him, it was… he told me, “I have rarely met people like that, in fact—people who have the desire and then fight to achieve something.”

During the exploration phase, participants mentioned institutional support as a significant aid for strategic career decision-making. Recalling how he chose when he was a teenager, Jean, 31, a former money courier, appreciated the career decision-making process initiated by his public disability insurance counselor:

What I think is cool is that when you’re at school, when you have a career counselor who says, “What do you want to do now?” I think for 90% of the kids, I saw with my buddies, we were not interested in the thing […] While now, when we elaborate plans and everything, […] I am a lot more mature, saying “Well, I have to do something I like; I still have X years to go, if not more, because the laws might change; I have to have a decent salary.” […] I think I’m even luckier because I’m facing this situation of career choice, but with maturity and with more valid criteria than “I want to go party on weekends.”

Finally, interviewees emphasized the support received during the implementation phase, which took the form of concrete internship opportunities and training courses leading to formal qualifications. Véronique, 44, a former hairdresser facing a chronic illness, appreciated that a professional offered to give her access to his network to help her find an internship position:

I’m going to be evaluated next week, in relation to the first month that’s gone by. I think my rate will increase slightly, and the goal is to continue this dynamic a little bit, and then I’m going to be followed up by a vocational rehabilitation coordinator who will, through his network, help me find an internship in a company.

### Negative Relational Influences

Negative relational influences cover all the moments of change and all the sources of influence. We divided these influences into 13 subthemes (see Table 3).

**TABLE 3:** Subthemes within negative relational influences.

	Leaving	Shifting	Exploring	Implementing
Personal environment	Misunderstandings and judgments (3) Loneliness (3)	Silence (6)	Tensions and imbalances (4)	Family strains (4)
Work environment	Disrespectful employers (8)			Labor market prejudices (7) Labor market rigidity (4) Difficulties in networking (4)
Institutions		Administrative slowness (4) Rigidity and constraints (4)	Inconsistent and inadequate support (5)	Inappropriate adult education programs (9)

*Numbers refer to frequencies of participants.*

#### Negative Influences From the Personal Environment

Misunderstandings and judgments during the leaving phase were the first form of negative influence coming from the personal environment. In this subtheme, participants reported that family and friends did not really recognize what they were going through and may have implicitly or explicitly criticized their inability to overcome their difficulties. A second negative influence occurring during the leaving phase was more indirect: It referred to the absence of potentially helping others within the personal environment, leading to feelings of loneliness. Instead of actual negative influence, some participants characterized some of their career change experiences as the unavailability of possible support from loved ones and their close entourage. Giuliana, 47, a former surgeon, shared her loneliness as follows:

I am alone here: no family. I have nothing, nothing, nothing, nobody. So, my only family are my colleagues, my patients, who meanwhile call me all the time: “When are you coming back?”

A more intentional form of loneliness characterized the shifting phase: Some participants decided to remain silent and isolated themselves to avoid sharing what they were living through. Henry, 58, a former humanitarian coordinator, adopted the strategy of avoiding discussion of his unemployment situation with his entourage. When asked whom he was talking to about this situation, it took him a long time before answering,

I don’t talk that much about [my career change] to be honest. It’s kind of something I’m keeping to myself, for now. I’ll see how it turns out and to what extent we’ll have to get into it.

The exploration phase goes hand in hand with tensions and imbalances in the private sphere, including intimate relationships. Kevin, for example, found it humiliating to earn less money and be unable to contribute equally to his activities with his partner:

When you go from a 100% salary to an 80% salary. well, there you go […]. That’s pretty hard, in the sense that you really feel like you’re a bit of a burden—well, not a burden, because I still pay the same bills […]. I really wanted to keep the same bills to tell myself that I don’t want to be a burden for the person I live with. But of course, the weight is still there […]. I don’t know, but when we want to go on holidays, I can’t necessarily put money aside like he does. So, if tomorrow he says to me, “OK we’re going on vacation, how much do you have set aside?” [stifled laughter] well, we won’t go very far. So, maybe I’ll feel a drag. I may not be able to afford to go to a restaurant, to offer to eat out […]. But it’s true that there’s still a weight on my shoulders. I think that I would have lived better if I lived alone. […] Maybe I also have a problem with being dependent on others, like being dependent on disability insurance or being dependent on unemployment or social assistance. It’s hard, you know.

Finally, the implementation phase was sometimes inhibited by family strains. In particular, family duties could prevent professional mobility and flexibility, or reduce financial resources. This was the case for Gabriel, 29, a former carpenter suffering from disabling back pain, who could not afford to be without a salary to attend an appropriate training course because he had to support his two children:

[The training programs I consider] are not big trainings, so it’s okay. I couldn’t leave, I don’t think I would have gone on a big 2- or 3-year course. It would have been… and then the family situation doesn’t really allow for that either. With a child at home, there’s a second one on the way, so it’s not…

#### Negative Influences From the Work Environment

Negative influences coming from participants’ work environments manifested at the beginning and at the end of the career change process. During the leaving phase, some complained about disrespectful former employers, who did not understand or consider their needs and rights. For example, Anna, 49, a former nurse, was forced to return to work while recovering from wrist surgery:

After the accident, I was pressured to go back [to work] and then it was almost mobbing […] Psychologically it was very, very, very, very hard. In the end I was even forced to contact a lawyer, because I was pushed up to the director. He proposed to me—I still had wires in my wrist after the second operation when they removed the pin—he wanted me to move on foot for the follow-up of the students, to do small works during the rehabilitation. And then the director, I explained that it’s too soon to ask that, and he said, “I’m willing to talk with your doctor.” They did, they tried to mob me.

While implementing their career plans, participants encountered three forms of negative influence coming from the work environment they tried to integrate: Half of them had to cope with labor market prejudices toward career changers; some faced rigidities in the labor market; and others struggled to create and enter professional networks, which is a hindrance to accessing interesting positions. Former library data manager Nancy, 41, mentioned experiencing a combination of prejudices associated with her gender, age, and origin:

I think it’s very difficult [to find a job], especially in Switzerland, or maybe it’s because I’m a woman, because of my age, because I’m a migrant, I don’t know… Because when you change [careers], you have to start again and maybe employers have to take a chance. But for me, with my experience, there are not many opportunities to change.

#### Negative Influences From Institutions

Two forms of institutional barriers were pointed out during the shifting phase. First, some participants stated that administrative procedures slowed down the change process, as if the institutional pace was too slow for their own pace of change. Second, participants suffered from rigid, constraining, rule-imposing institutions that made them feel out of control of their career changes. Beatriz stated the following concerning the rules of the public disability insurance:

You have to tell everything: if you fart, you have to justify why. You want to take a vacation? You have to say, “I’m going on vacation from such and such a date to such and such a date.” You have to say, “I’m sick,” you have to say, “I was sick on such and such a day,” and after a while, that’s it. You get your salary on the third of the month, while everyone else gets their salary on the 25th. So yeah, it’s full of little constraints like that, which I don’t like. So, disability insurance is not for me.

During the exploration phase, institutional support was sometimes considered inconsistent or inadequate. For example, Giuliana was initially forced to abandon her plans to switch from being a surgeon to working as a medical diagnostician. After being forced to close her practice, public disability insurance still allowed her to continue consultations despite its initial decision:

After the [disability insurance’s] medical advisor waited 3 months to answer, and then he received the report of the specialist—the hand surgeon—well, he told me, “Now you can continue with the consultations.” Well done [applauds]: First you make me close [my practice], and now I have to continue… how?

When it came to implementing a career plan, adult education programs appeared rigid and not adapted to participants’ needs and means as possible adult learners. For example, Louis, 46, a former airline CEO, was not able to access the university, despite his career being relevant to the targeted degree:

I considered [a new training]. I went to the University of [Swiss city] to do an MBA and then they told me, “But sir, you don’t even have a university degree, so you don’t have access to our MBA. You are not eligible for this MBA program, even if you do have an interesting professional background.”

### Ambivalent Relational Influences

Four subthemes reflected the way in which some influences (Table 4).

**TABLE 4:** Subthemes within ambivalent relational influences.

	Leaving	Shifting	Exploring	Implementing
Personal environment		Benevolent but inappropriate support (7)		
Work environment	Ambivalent exit from former job (3)			
Institutions		Appreciated help, but rigidity, and feared dependency (7)		Partly imposed, partly chosen career plan (6)

*Numbers refer to frequencies of participants.*

#### Ambivalent Influences From the Personal Environment

Ambivalences in the personal environment appeared during the shifting phase. Half of the participants reported that their entourage was supportive in benevolent ways; but this support was often inappropriate, clumsy, and led to misunderstandings. Marie, 42, who lost her job as a bookseller, stated that her friends’ suggestions for her career change were inaccurate and inappropriate, because they had an incorrect image of her skills and contrasting views on the future of her employment sector:

Sometimes they make projections about things that I should be doing and I don’t feel up to it. For example, for me it’s quite heavy, this story of having done a master’s degree gives the impression that I have a great qualification. But in fact, I feel rather handicapped by that, because I was a rather weak student [laughs]. In general, they offer their support, they encourage, they sympathize and then when they have ideas, they share them, but there is no. well, their opinions on the future of bookselling… Sometimes there are people who say, “forget it, there’s no chance.”

#### Ambivalent Influences From the Work Environment

Within the work environment, participants mentioned ambivalence when they had to leave their former jobs. For example, Kevin’s former boss did not want to accept that a performing and appreciated employee like him had no choice but to leave:

My boss came back, and then I said, “I’m going to start really going to a doctor to find out what’s causing this [allergy], because right now I can’t stand it.” So, I really warned him, but did he really get the idea that I was going to quit overnight? I don’t know, I think there was a bit of a denial. I was kind of […], the good collaborator.

#### Ambivalent Influences From Institutions

Institutional influences manifested in ambivalent ways both during the shifting process and when it came to implementing a new plan. During the shifting phase, half of the participants noted that although they cherished being helped, they were afraid of rigidity or becoming too dependent on these supports. William pointed out the rigidity and unsuitability of institutional help when changing occupations. At the same time, he appreciated the networking opportunities it had given him, though:

I think [state unemployment support] does not evolve enough. At the same time, my counselor gave me courses that gave me opportunity to meet people, so I’m not going to completely criticize unemployment […] There are also good points, positive sides […]. On the other hand, it’s still very traditional, like just doing courses on how to improve in interviews. For situations of career changes, there is no action—no support.

Participants also stated that they encountered ambivalent institutional influences when trying to implement their career changes. For some, the new career plan was neither totally freely chosen, nor completely imposed by the institutions supporting them. Jean, for example, learned tardively that he could be financially supported for new training, but also stated that this support was not unrestricted:

At the beginning I had a lot of questions. I didn’t always know what I could afford to do. The misdirection was related to this lack of information, because basically I didn’t know what I was entitled to from the beginning. […] I’m an ex-money courier: what can I do? Can I expect to end up as an engineer or a chemist? […] I’m realistic, let’s say. I’m not going to go to [the integration foundation] and say that I’m interested in chemistry […]. Depending on the income you have [i.e., the former job’s pay], [the public disability insurance] unlocks substantial means. Unfortunately, this is the information that I had to go and get […]. So, my choice is wider too. That’s why I limited myself. I didn’t feel limited, it was reasonable. It’s not like you can pretend to go to university, to take another high school diploma, etc.

## Discussion

The general aim of this study was to understand how the social environment influences the process and experience of involuntary career changes. We split this into two specific objectives: (1) identifying the sources of relational influences and (2) understanding how these relational influences affect the process and experience of involuntary career changes. Addressing these objectives is critical because research shows that work transition processes are shaped by complex, yet underexplored, relational influences (e.g., Motulsky, 2010), and because recent theoretical perspectives stress that relationships profoundly shape career paths (Kenny et al., 2018).

A first general observation that emerged from our findings is that relational influences on career change processes were highly heterogeneous and differentiated, underlining the inherent complexity of relational influences on careers (Kenny et al., 2018). On the one hand, we identified a wide range of subthemes (i.e., 10 positive, 13 negative, and five ambivalent influences) covering diverse sources of relational influences at distinct moments in the transitional process. This finding provides empirical support for the need to move beyond a static and dualistic view of contextual influences on careers, which consists of splitting them into supports and barriers or into positive and negative influences (see Sheu and Bordon, 2017). Instead, relational influences take multiple forms, are fluid, and sometimes have mixed effects.

On the other hand, only three out of 28 subthemes concerned more than half of the participants; namely, emotional support from the personal environment and employers’ disrespect during the leaving phase (nine and eight participants, respectively) and inappropriate adult education programs during the implementation phase (eight participants). The sources and types of relational influences, then, seemed to depend on each participant’s specific context and unique work and life path.

We can provide several more targeted observations related to our specific study goals. In the following sections, we discuss these observations in detail, highlight their importance in understanding the career change process, and underline some limitations and practical implications stemming from our research.

### Who Others Are

Our first specific research goal was to identify the sources of relational influences on involuntary career change processes and experiences. We identified three sources of influence: the personal environment (including family members and close relationships), the work environment (including former and current employers and companies), and institutions participants interacted with to obtain support for their career changes (mainly the disability and unemployment insurance bureaus). Several cross-cutting observations allow us to discuss each of these sources.

#### The Alternating Influences of Personal Environment

Influences from family, friends, and partners tended to alternate between positive and negative. During the shifting and implementation phases, the personal environment exclusively had a negative impact on participants, leading career changers to prefer not sharing their change experiences and restraining their projects. In contrast, during the leaving and exploring phases, family and friends both positively and negatively affected career changers. Positive influences mainly took the form of emotional support, while negative influences manifested through diverse sorts of relational stress due to misunderstandings, imbalances, tensions, or negative judgments from close relatives. The effect of the personal environment might then vary depending on the moment when others intervene. Such a finding stresses the importance of taking into account the temporal and processual dimensions of career changes (Kenny et al., 2018). It also indicates that, although close relatives might be supportive when people learn they have to change careers and when they reflect on the direction this change may take, close relatives might be less helpful during the in-between phase and when it comes to concretely implementing a new career plan. Beyond their temporal nature, alternating influences from the personal environment also highlighted the complex, sometimes ambivalent, and intricate web of interpersonal relations. Adding to what has been shown by most existing research (e.g., Blustein et al., 2013), our results suggest that influences from the personal environment might include a “dark side,” which calls for the benefits of environmental supports to be put into perspective. Overall, our results confirmed that people’s life domains are highly interrelated. Working experiences and career decisions partly depend on—and influence—what people experience in their personal life spheres (Kenny et al., 2018; McMahon and Patton, 2019).

#### Hostile Work Environments

Obviously, influences from the work environment operated at the beginning and at the end of the career change process; that is, when participants learned that they had to quit their former jobs and prepare for change (i.e., the leaving phase) and when they strived to move to a new occupation (i.e., the implementation phase). These influences were predominantly negative, whether because former employers complicated the transition and prevented a smooth exit, or because several types of labor market obstacles (e.g., rigidities and prejudices) threatened the new career plan. Within our study’s context, this result could indicate that the labor market—particularly, some companies and employers—was either hostile toward career changers or not ready to consider their specific challenges and needs. Combined with the observation that national statistics on this issue are imprecise and rudimentary (Office Fédéral de la Statistique [OFS], 2020), our results may reflect a general lack of knowledge and familiarity with career changes in the Swiss context.

#### The Critical Role of Institutions

Institutions deeply affected participants’ career change processes and experiences. Their prevailing influence took multiple forms during the shifting stage (i.e., through seven distinct subthemes), which is not surprising given that this is the stage at which institutions are most solicited to support workers’ transitions. Institutional influences were both positive and negative. Interestingly, recognition emerged as a critical positive element. Some participants valued the fact that professionals acknowledged the complexity of their situations and the difficulties they faced. This finding might show the importance of the recognition of the emotional and social challenges encountered by people who struggle with their careers beyond the weight of instrumental (e.g., financial) support. It is then consistent with previous research outlining the crucial role of social recognition on careers, such as Blustein et al.’s (2013) study, which showed that the experience of unemployment is less detrimental when people benefit from emotional support. Conversely, Howes and Goodman-Delahunty (2014) showed that a lack of recognition can lead to a decision to change occupation. However, several negative effects of institutions were also pointed out, broadly stressing that institutional supports were often considered inappropriate. This inappropriateness was mainly the result of a mismatch between participants’ needs and the support actually provided by the institutions. For example, when flexibility was requested, rules were rigid; when quick action was expected, procedures were slow; and when freedom of choice and action would have been beneficial, participants were faced with constraints.

### What Others Do

Our second specific goal was to understand how these sources of relational influences affected the involuntary career change process. Our results indicated that career changes can be considered both as a highly social and a rather lonesome experience, and that ambivalent relational influences deserve particular attention.

#### Career Change as a Social Experience

The career change experiences participants shared were socially shaped. Others constantly gravitated around them and influenced how participants approached and experienced the change process. On one hand, positive relational influences were perceived at each stage of the career change process and took multiple forms. No single type or source of influence was prominent, which confirms the uniqueness of every single career change experience and life course. On the other hand, the same applies for negative relational influences, with one noteworthy exception: As mentioned in the previous section, the inappropriateness of institutional supports pervaded the narratives of several participants and discouraged them from benefiting as much as they might have otherwise. These results confirmed the relevance of the first tenet of the relational perspective on work and careers (Kenny et al., 2018), which suggests that working is a fundamentally relational experience and that relationships can have both positive and negative effects on people’s working lives. In contrast with this tenet, however, our study showed that negative relational influences were not limited to the workplace but could spread beyond the work setting to include institutions and the private sphere. These findings also corroborated the results of past research on career transitions, such as Motulsky, 2010, who showed that relational processes at diverse levels can both enhance and hinder midlife women’s career transitions.

#### Career Change as a Lonesome Experience

If career change experiences were deeply relationally embedded, they were not necessarily socially shared by career changers. Indeed, the change process was also characterized by moments of isolation and withdrawal, during which people managed or wanted to manage some of the challenges of career change on their own. Three forms of loneliness resulted from our analyses, which we refer to as unintentional, deliberate, and experiential. *Unintentional loneliness* refers to moments when the absence of possibly helping relationships became a burden—for example, a lack of networking or of benefiting from emotional support. In contrast, at other moments loneliness was *deliberate*: Participants purposely decided to isolate themselves and not share their difficulties in order to feel normal, not perceived as needy, possibly in an attempt to protect their self-esteem. Finally, *experiential loneliness* resulted from the potential effects of relational barriers on participants. Not feeling understood by family and friends, perceiving resistance in the labor market, and witnessing the rigidity of transition and education programs may have implicitly signaled to them that they were alone in their career change journeys. This experiential loneliness mirrors the social disconnectedness experienced by unemployed people (Blustein et al., 2013). Overall, these results confirmed that “relational influences can, at times, present considerable challenges to individuals negotiating work-based tasks” (Blustein, 2011, p. 9).

#### Multilevel, Multiform Ambivalence

Our findings indicated that ambivalence was displayed at all levels of relational influences, whether through caring but awkward relatives, rigid institutions with supportive professionals, or companies that participants both regretted working for and were happy to leave. In addition to the specific and situated ambivalent situations highlighted in our analyses, it seems legitimate to assume the existence of another form of ambivalence associated with the processual and temporal dimension of career changes. *Temporal ambivalence* would thus add to *situated ambivalence* to indicate that relational influences can fluctuate throughout the change process: The same relationship may have positive influences at one point in the process but become marginal or even negative at another point. This appeared to be the case, for example, with close friends and family, who were typically mentioned as important sources of emotional support during the leaving phase but were ignored or avoided during the shifting phase. Overall, the incidence of multilevel and multiform ambivalent relational influences tended to emphasize the complexity and mutability of relational processes. These findings echo Motulsky’s (2010) results about women in career transitions, stating that women’s relationships “included both connections and disconnections within the same person” (p. 1100). Overall, our findings complement and expand upon previous research and theories of career development (e.g., Blustein, 2011; Sheu and Bordon, 2017), which have portrayed a polarized and rather static understanding of others’ influences on careers.

### Limitations and Perspectives

Our study had four limitations, leading to some research perspectives. First, we opted for a horizontal analysis, searching for common themes and subthemes across participants (Braun and Clarke, 2006). This implies omitting the biographical, within-person dimension of career change experiences. As we observed that each experience is unique and that relational influences seemed to involve a temporal dimension, future research should focus on the longitudinal feature of career changes. It would thus be relevant to implement a qualitative longitudinal design that combines within- and between-case analyses (Neale, 2021).

Second, our recruitment procedure involved reaching out to institutions that support people in transition. It is therefore likely that the participants’ accounts focused on the role played by these institutions, which could in part explain the prominence of this source of relational influence in our findings. The recruitment procedure also prevented us from accessing the experiences of career changers who were not institutionally supported and who might therefore have had to cope with even more marginalizing transitional challenges. Future research could then implement alternative sampling strategies to access less institutionalized involuntary career change experiences.

Third, at the time of the interviews, not all participants were necessarily at the same point in their career change processes, which may have shaped their narratives of their transitional experiences. For example, the retrospective view of the change process might have been more positive for those who already had a specific, achievable project when interviewed. In contrast, more pessimistic views might have been expressed by participants who had not yet identified meaningful and reachable career opportunities. Again, qualitative longitudinal studies could be pertinent to consider this aspect.

Fourth, we did not clearly detect distal relational influences on career change experiences (e.g., opportunity structures, macrocontextual, cultural, or societal effects; see for example Sheu and Bordon, 2017), probably because these influences are less tangible and more difficult for participants to recognize. Nevertheless, as suggested in the fifth tenet of the relational perspective on work and careers (Kenny et al., 2018), distal influences are not to be neglected, and we observed hints of these types of effects in some subthemes. For example, labor market prejudices and rigidities, as well participants’ choice to keep silent in order to feel normal, suggested the existence of cultural and societal forces detrimental to the transition experience. However, in this study, these influences were only speculative and suspected; thus, further studies could address them in a more targeted manner.

### Practical Implications

“Uncertainty also carries the potential to augment the need for affiliation and sensitivity to relatedness” (Flum, 2015, p. 147). Because involuntary career changes involve a high degree of uncertainty and create major discontinuities in people’s lives, nurturing the relationships of those who experience them becomes critical. Based on our results, this relationship-building work can be implemented at the labor market, institutional, and personal levels. At the labor market level, it would be relevant to raise awareness among labor market stakeholders about the specific needs and issues of involuntary career changers—for example, focusing on the fact they have to cope with undesired events in their lives. Interventions targeting employers and companies could also inform about and advocate for respecting workers’ rights and duties. At the institutional level, professionals supporting people who are forced to change careers should be sensitized to the need of implementing adaptable interventions that suit their challenges in terms of rhythms and specific needs, among other factors. Another recommendation at this level would be to foster tertiary and vocational trainings tailored to an adult population, with special consideration for possible family and financial constraints. At the individual level, it seems crucial to avoid worsening career changers’ loneliness and silence by identifying adequate supports. Interventions should help career changers recognize who is best able to help, how, and when. Members of the personal environment could be involved in these interventions to help them become effective helpers instead of benevolent barriers.

## Conclusion

Our study showed that involuntary career changes are deeply shaped by relational influences. These influences are multifaceted, sometimes manifesting as resources that support the process of change, while in other cases as barriers that hinder it. Between these two poles, other influences are rather ambivalent, meaning that they can be both appreciated and avoided, or that their effects are ambiguous. Moreover, a temporal dimension must be considered when trying to understand relational influences. Influences can come at the right time or at the wrong time, and their effects can fluctuate depending on the transitional phase a person is in. Among the sources of relational influences, institutional influences are omnipresent and have the power to facilitate or constrain career change processes. This depends on institutionalized programs’ appropriateness to the specific and situated needs of each person. Ultimately, while they result in an eminently social experience, involuntary career changes are also marked and framed by moments of loneliness. These moments reflect an inadequacy of available supports, but also a sense of shame that individuals may feel toward taking advantage of them or being labeled as career changers. As a result, it seems imperative both to identify and strengthen resourceful others and to better identify and grasp situations of loneliness. Indeed, these situations can prevent people from taking advantage of possible supports; make the experience of change more insecure, if not traumatic; and compromise the success of the career change process.

## Data Availability Statement

The datasets presented in this article are not readily available because they are subject to restrictions imposed by the funding institution and ethics committee. Requests to access the datasets should be directed to JM, jonas.masdonati@unil.ch.

## Ethics Statement

The present study was reviewed and approved by the Ethics Committee of the Faculty of Social and Political Sciences of the University of Lausanne (project number C_SSP_052021_00003). The participants provided their written informed consent to participate in this study.

## Author Contributions

JM and CF carried out the analyses. JM wrote the manuscript with support from CF and MP. All authors contributed to the article and approved the submitted version.

## Conflict of Interest

The authors declare that the research was conducted in the absence of any commercial or financial relationships that could be construed as a potential conflict of interest.

## Publisher’s Note

All claims expressed in this article are solely those of the authors and do not necessarily represent those of their affiliated organizations, or those of the publisher, the editors and the reviewers. Any product that may be evaluated in this article, or claim that may be made by its manufacturer, is not guaranteed or endorsed by the publisher.

## Appendix: Interview Guideline

(1) Sociodemographic information

Age
Living situation
Family situation
Education level
Country of origin

(2) Career path

Can you tell me what occupations you have held throughout your career, and how long they lasted?
In the past, what made you change jobs?
What life events have affected your career path?
Here you can see images (Reitzle et al., 2009) that represent career paths. Which image would best describe the evolution of your career path? Which image best describes your sense of control over your journey?
How satisfied were you in your last job?
What is your current professional situation?

(3) Career change process

Can you tell me what brought you to this point professionally?
What are the reasons for your career change?
How has the pandemic situation influenced the transition process?
To what extent do you feel that you have been able to choose this career change?
To what extent did you expect this career change?
What is the likelihood that you will be able to return to your previous occupation?
What did you say to yourself when you learned that you had to change careers?
What did you feel at that moment?
What links do you perceive between the job you had before and your current career plan?
Could you please complete the following sentences? “What I’m going to regret about my former profession is _____”; “What I’m glad I no longer have is _____”; “What I fear in my new profession is _____”; “What I’m looking forward to in my new profession is _____”
How do you feel about changing occupations/leaving your occupation/entering a new occupation?
How important is this change in your life?

(4) Identity

Which occupation do you identify with? Can you explain this choice?
How did you choose your new occupation?
To what extent does this new occupation suit you?
How does your career change also change how you see yourself as a person?
More generally, what roles define you today?
How does your career change influence these roles?
To what extent has this career change had repercussions on those around you?
With whom do you discuss your career change?
How do you talk about this situation to people around you?
How do these people react when you talk to them about your career change?
What are people’s perceptions of you as you undergo career change?
Try to project yourself into the distant future, for example, 10 years from now. Who do you think you will be at that moment professionally? Still in about 10 years, what would your ideal situation be, and what situation would you like to avoid?

(5) Resources and barriers

What resources facilitate help you cope when facing the challenges of a career change?
What do you have, or what would you need, to help you in this transition?
What is standing in your way?

(6) Relationship to work

How important is work in your life?
If you won the lottery, would you still work? For what reasons, and under what conditions?
What puts you in a good mood in the morning, anticipating a day at work? To what extent was this the case at your previous job?
How has your career change affected the importance and meanings you attach to work in your life (if at all)?

(7) Relationship to training (when pertinent)

How do you feel about entering a new training program?
What expectations do you have of the training process?
What role does training play in your career change?
How do you feel about undertaking a new learning process?
